# Data on the radiometric survey over a kaolinitic terrain in Dahomey Basin, Nigeria

**DOI:** 10.1016/j.dib.2018.03.088

**Published:** 2018-03-26

**Authors:** T.A. Adagunodo, O.S. Hammed, M.R. Usikalu, W.A. Ayara, R. Ravisankar

**Affiliations:** a Department of Physics, Covenant University, Ota, Ogun State, Nigeria; b Department of Physics, Federal University of Oye Ekiti, Oye Ekiti, Ekiti State, Nigeria; c Post Graduate and Research Department of Physics, Government Arts College, Tiruvanamalai 606603, Tamilnadu, India

**Keywords:** Thorium, Potassium, Uranium, Tiles, Dahomey Basin, Ifonyintedo, Dose rate, Kaolin

## Abstract

This article consists the in situ data sets of activity concentrations of radionuclides (K-40, Th-232 and U-238) and gamma radiation dose rates measured about 1 m above a kaolinitic terrain in Ifonyintedo, Dahomey Basin, SW Nigeria. Nineteen (19) data points were randomly occupied at the lower axis of the kaolin field using a hand-held detector known as Super-Spec (RS 125). At each data point, the measurements were taken four times, while their averages and standard deviations were estimated in order to ensure accuracy. The radiometric survey was carried out between December, 2017 and January, 2018. The data sets were processed and analyzed via a descriptive statistics. The data can be explored further by estimating the radiological risks to the miners on the field, and to correlate the activity concentrations of the data with the activity concentrations of the tiles that are produced from the kaolin deposits in Ifonyintedo. Furthermore, the data from this article could be compared with other data acquired over a kaolinitic terrain across the globe.

**Specifications Table**

**Table:** 

Subject area	*Geophysics*
More specific subject area	*Environmental Geophysics*
Type of data	*Table and figure*
How data was acquired	*Super-Spec hand-held Spectrometer (RS 125), Global Positioning System*
Data format	*Raw and analyzed*
Experimental factors	*Radiometric measurements were conducted randomly across a kaolin deposit field in Ifonyintedo using Super-Spec (RS 125)*
Experimental features	*Activity concentrations of Potassium (*^*40*^*K), Thorium (*^*232*^*Th), and Uranium (*^*238*^*U),as well as Dose Rates of all occupied locations were determined*
Data source location	*Ifonyintedo, Ipokia Local Government, Ogun State, Nigeria*
Data accessibility	*All the data are in this article*

**Value of the data**

• The data could be used to estimate the radiological risks associated with the overexposure of the miners to the radionuclides on kaolin deposit field.
• The method employed here can be replicated on kaolin deposit field across Nigeria for radiological assessment on miners, and correlation between the kaolin's activity concentrations, and the radioactivity concentrations in tiles that were produced from the deposits.
• For educational purposes in courses such as: geophysical field works, ground radiometric method, and radiological hazard assessment. Some of the recent data articles of this format can be explored in [1], [2], [3].

## 1 Data

The data sets contain the in situ measurement of K-40, Th-232, U-238 and the gamma dose rates over kaolin deposits in Ifonyintedo, Dahomey Basin, SW Nigeria. The data were randomly occupied at the lower axis of the field from nineteen (19) locations as presented in Table 1. The coordinate and elevation of each location were determined with the aid of global positioning system (GPSMAP 78). All soils are radioactive as a result of cosmogenic or and primordial radionuclides being available naturally. Hence, the products or materials that are derived from these soils are radioactive. Exposure to more than required activity concentrations and the gamma dose rates have been attributed to some acute health problems such as: “Chronic lung diseases, mouth necrosis, anemia, acute leucopoenia, teeth fracture, cataract, cancer, hepatic failure and leukemia” [1]. The statistical analyses were further carried out on the data sets for further exploration.

**Table 1:** Activity concentrations and dose rates of each location in a kaolinitic terrain.

Sample	U-238 (Bq/kg)	Th-232 (Bq/kg)	K-40 (Bq/kg)	Dose rates (nGy/h)	Longitude	Latitude	Elev. (m)
LA1	37.05±0.02	65.77±0.24	187.8±3.12	63.38±6.36	002° 47.567′ E	006° 46.094′ N	89
LA2	28.41±0.01	75.92±0.30	156.5±3.53	64.39±0.84	002° 47.572′ E	006° 46.092′ N	87
LA3	48.17±0.01	73.08±0.22	125.2±4.28	70.00±1.95	002° 47.570′ E	006° 46.089′ N	83
LA4	116.09±0.02	77.95±0.13	93.9±6.99	101.22±5.50	002° 47.569′ E	006° 46.083′ N	90
LA5	43.23±0.01	77.14±0.11	125.2±3.24	70.27±9.20	002° 47.567′ E	006° 46.077′ N	89
LA6	51.87±0.03	73.49±0.26	156.5±1.94	73.16±1.21	002° 47.567′ E	006° 46.072′ N	83
LA7	11.12±0.03	58.06±0.35	156.5±0.22	46.15±4.60	002° 47.568′ E	006° 46.066′ N	86
LA8	24.70±0.03	62.52±0.14	93.9±0.36	52.14±2.84	002° 47.572′ E	006° 46.068′ N	88
LA9	34.58±0.01	68.21±0.20	125.2±1.49	61.15±4.11	002° 47.576′ E	006° 46.072′ N	86
LA10	53.11±0.02	60.49±0.17	62.6±4.21	62.00±6.90	002° 47.576′ E	006° 46.074′ N	89
LA11	43.23±0.01	76.73±0.39	125.2±0.55	70.03±3.20	002° 47.575′ E	006° 46.078′ N	89
LA12	53.11±0.01	98.25±0.18	187.8±0.43	89.84±1.10	002° 47.573′ E	006° 46.076′ N	91
LA13	39.52±0.02	90.13±0.26	31.3±0.38	72.53±1.50	002° 47.570′ E	006° 46.076′ N	88
LA14	37.05±0.02	73.49±0.35	31.3±0.65	61.48±5.36	002° 47.560′ E	006° 46.060′ N	91
LA15	58.01±0.01	76.73±0.15	62.6±2.68	73.88±4.95	002° 47.560′ E	006° 46.077′ N	91
LA16	54.34±0.03	59.68±0.26	93.9±1.50	63.36±4.55	002° 47.539′ E	006° 46.059′ N	89
LA17	40.76±0.01	65.37±0.33	31.3±1.00	58.23±0.60	002° 47.533′ E	006° 46.054′ N	89
LA18	61.75±0.01	62.93±0.11	93.9±0.24	68.53±1.19	002° 47.533′ E	006° 46.075′ N	86
LA19	50.64±0.01	67.40±0.11	125.2±2.50	67.67±1.21	002° 47.555′ E	006° 46.073′ N	86

## 2 Experimental design, materials and methods

Kaolin is one of the mineral resources that are available in commercial quantity in Nigeria. Kaolin is one of the types of clay found in nature, with the chemical composition of Al_2_Si_2_O_5_(OH)_4_. Its economic importance are found in plastic, paper, ceramics, food additives, cosmetics, paint, medicine, agriculture, construction and cement industries. The main component in ceramic tile body is kaolin clay. In construction industry, all the available raw materials derived from soils or and rocks are products of kaolin, clay, limestone, gypsum and pumice, which contain natural radionuclides such as U-238 and Th-232 and their decay series as well as the radioactive isotope of K-40 [4]. As a result of the chemical, physical and mineralogical composition of clays, they are considered in the production of refractories and ceramics including tiles, bricks, cements and concrete blocks. About 85–95% of kaolinites (formation as a result of weathering from kaolin) are present in these finished products. Quartz, feldspar, mica and illite are also contained in kaolin in lower proportion. Recently, it was emphasized that parts of the raw materials used for tiles are byproducts of decomposed-granites and phosphogypsum, which are radioactive in nature. Decomposed-granite is group of rock that takes its origin from granite through weathering such that the parent rock fractures into weaker rocks of smaller sizes. In addition, weathering yields material that easily crumbles into a mixture of gravel-sized particles known as grus, which later produces mixture of clay and silica sand or silt particles. Production of decomposed-granite varies, because their propensities to weather during weathering vary from type to type. Hence the unavoidably high activity concentrations and dose rates in the manufactured materials from raw material such as kaolin [5]. In must be reiterated that radiation emanates through cosmogenic, primordial, and anthropogenic sources. Distributions of the first and the last sources are insignificant to our environment. Primordial is the major radiation source that is very present in the earth and its environs. It is available in various geological formations as well as their daughters. Basic knowledge about the radionuclide distributions and radiation levels in an environment is crucial in evaluation of its effects to human being and race.

### 2.1 Study area

**Fig. 1:**
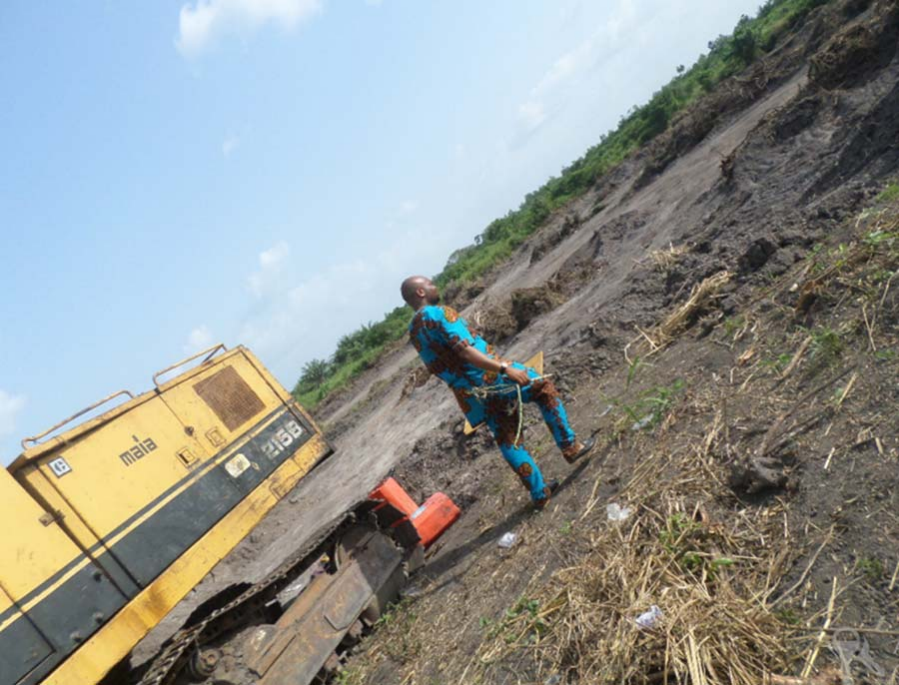
Lower axis of kaolin deposit field in Ifonyintedo.

**Fig. 2:**
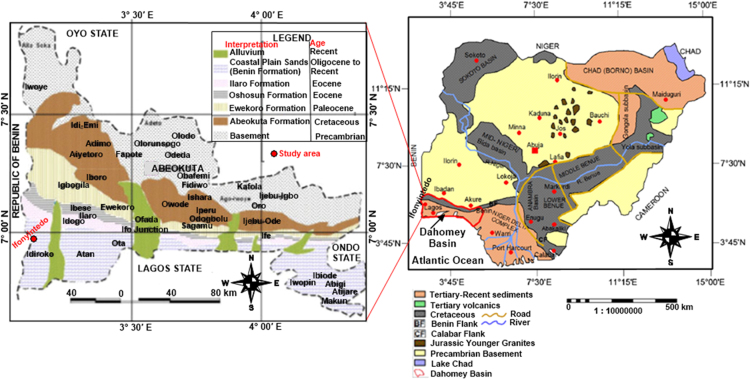
Geology of Nigeria revealing the geological domain of Ifonyintedo, Ogun state.

The study area is bounded by latitude 006° 46.054′ to 006° 46.094′ north and longitude 002° 47.533′ to 002° 47.576′ east. The elevation above the sea level ranged from 83 to 91 m, with an average of 88 m. nineteen (19) data points at the lower axis of the field. The mined kaolin in the study area is shown in Fig. 1. Ifonyintedo is a town located in Idiroko local council development area, Ipokia local government area, Ogun state, SW Nigeria. The town has a population of approximately 10, 000. The residents along Ifonyintedo axis are into farming and cottage industry. The major cultivated crops in Ifonyintedo include: cassava, maize, vegetable, and cash crop such as palm tree. The major cottage industries are cassava and palm oil industries. Recently, the discovery of kaolin deposits in commercial quantity has attracted the miners to Ifonyintedo. Commercial activities in Ifonyintedo have been improved greatly, due to its propinquity to the Republic of Benin's border. Like other suburbs of the study area, Ifonyintedo has a tropical climate, with distinct two seasons: rainy and dry seasons. Averagely, the rainy season span from March to November, while the dry season fluctuates from November to March, except on some minor cases where the rainfall is scarcely experienced between December and January. The mean temperature of the study is 26.5 °C. Further information about Ifonyintedo can be found on [6]. Nigeria is on the Pan-African mobile belt, which separates Congo Cratons and West Africa. The two major geological formations that spread in equal proportion are the sedimentary Basins (Upper Cretaceous in age) and Basement rocks (PreCambrian in age). Some of the studies from the Basement complex and Sedimentary Basin can be found in Refs. [7], [8], [9], [10], [11], [12], [13]. Ifonyintedo is grouped into coastal plain sands or Benin Formation on the geological map of Dahomey Basin (Fig. 2). Its age ranged from Oligocene to Recent. The Dahomey sedimentary basin extends from the eastern part of Ghana, Togo, and Republic of Benin to the western margin of the Niger Delta. The eastern half of the basin lies within Nigerian territory. The base of the basin comprised of unfossilerous sandstones and gravels weathered from the underlying Precambrian basement. The vegetation of the study area has given way to fens and other water loving shrubs and herbs.

### 2.2 Materials and methods

in situ measurements of activity concentrations of K-40, Th-232, U-238 and the gamma dose rates were taken over kaolin deposits in Ifonyintedo, Dahomey Basin, SW Nigeria. The data were randomly occupied at the lower axis of the field from nineteen (19) locations using a hand-held detector known as Super-Spec (RS 125). The coordinate and elevation of each location were determined with the aid of global positioning system (GPSMAP 78). The radioactivity measurements were taken four times at each location while their averages and standard deviations were estimated in order to ensure accuracy. The radiometric survey was carried out between December, 2017 and January, 2018. The detector used was manufactured by Canadian Geophysical Institute. It has high accuracy with probable measurement errors of about 5%. It offers an integrated design with a large detector, direct assay readout, data storage and high sensitivity. The assay mode of RS-125 Super SPEC provides sample concentration analysis with direct data display of potassium (K) in percentage (%), uranium (U) in part per million (ppm) and thorium (Th) in part per million (ppm). It uses sodium iodide (NaI) crystal doped with thallium [Tl] as activator. The approximate linear energy of the detector falls between 0.80 and 1.2 MeV, this range covers the majority of significant gamma-ray emissions from terrestrial sources. The detection of gamma-ray from cosmic ray is negligible due to the detector's low response to high-energy gamma radiation [14]. The recorded activity concentrations of K-40, Th-232, U-238 from the detector were converted to Becquerel per kilogram (Bqkg^−1^) in accordance with the conversion factor of International Atomic Energy Agency [15] and Omeje et al. [16].

### 2.3 Statistical analysis

**Fig. 3:**
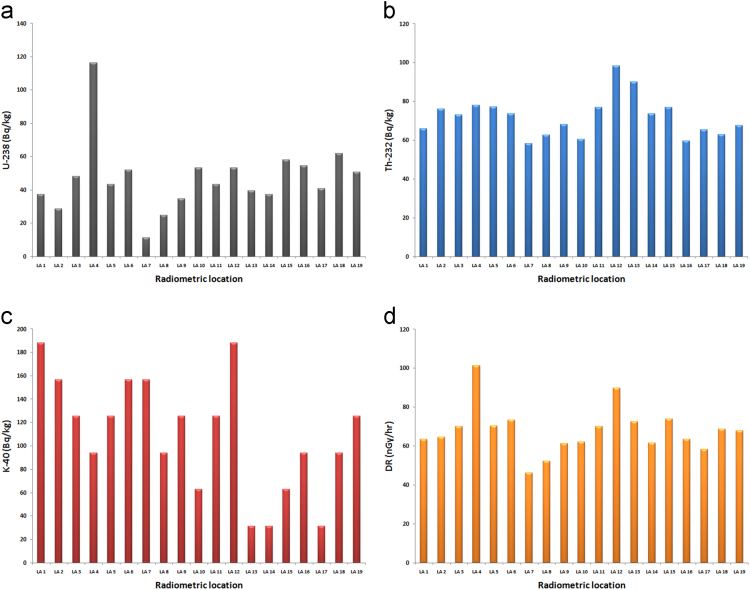
a. Bar chart of U-238 distributions. b. Bar charts of Th-232 distributions. c. Bar chart of K-40 distributions. d. Bar chart of dose rates’ distributions.

**Fig. 4:**
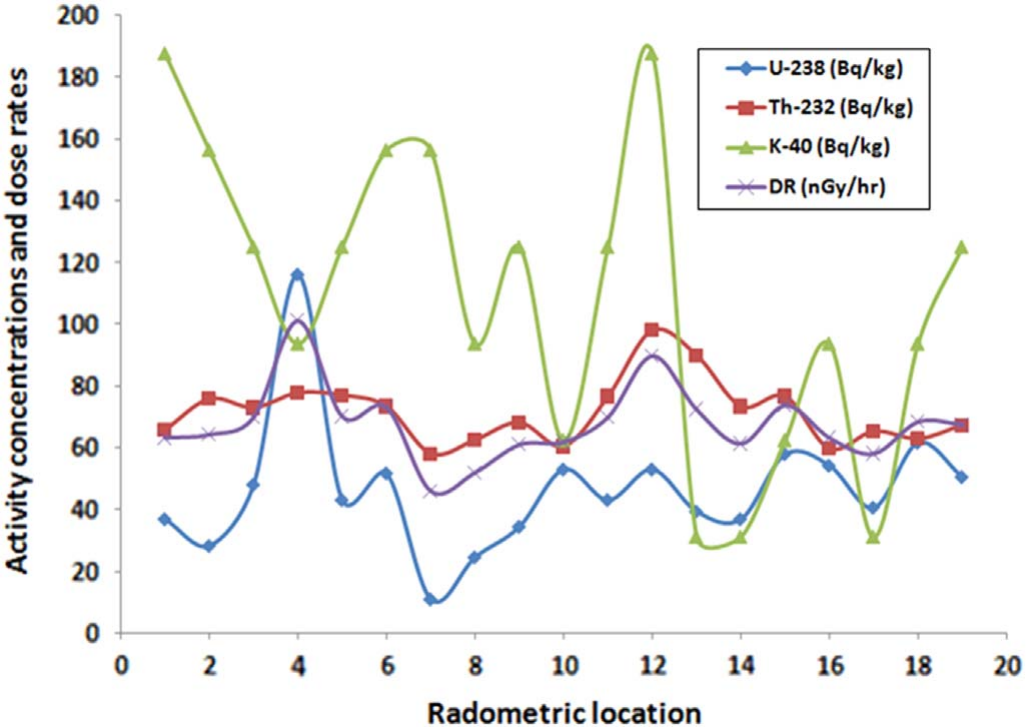
Comparison plot of the activity concentrations of radionuclides and the dose rates.

**Fig. 5:**
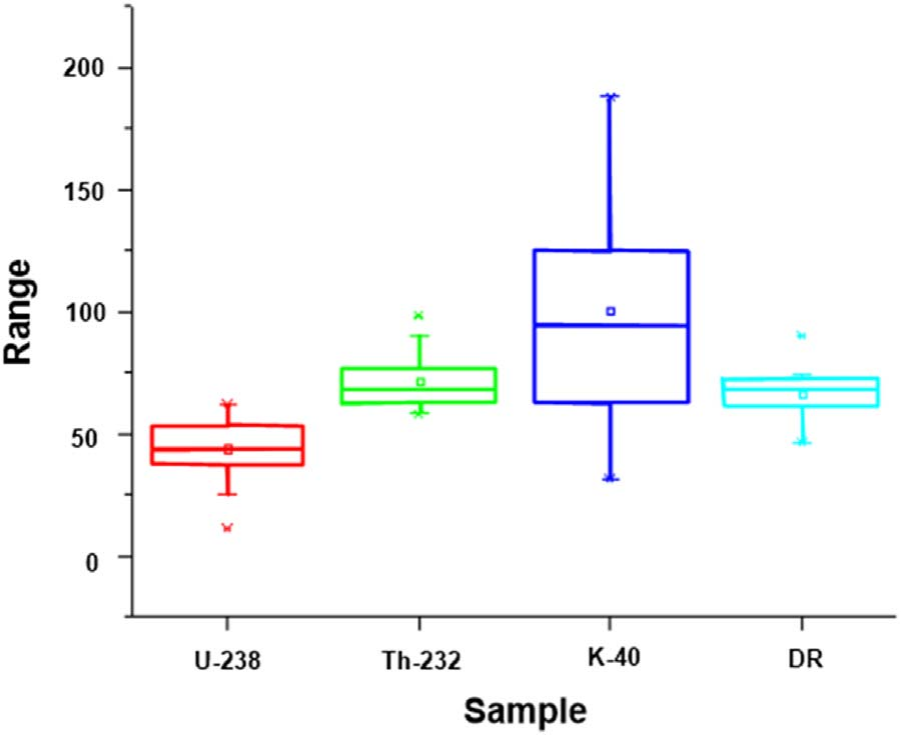
Box plot showing the comparison among the analyzed parameters.

Descriptive statistics for the measured gamma radiation and dose rates were presented in Table 2a, Table 2b. The estimated parameters are: total number of population (∑N), mean, standard deviation (SD), standard error of mean (SEM), variance, sum, skewness, kurtosis, uncorrected sum of squares (USS), corrected sum of squares (CSS), coefficient of variation (CV), mean absolute deviation (MAD), geometric mean (GM), geometric standard deviation (GSD), mode, sum of weights (SW), minimum, index of minimum (Imax.), 1^st^ quartile (Q1), median, 3^rd^ quartile (Q3), maximum, index of maximum (Imax.), Interquartile range (IR), and range respectively. The bar chart distributions of individual measured parameters were shown in Fig. 3a–d, while the comparison description of uranium, thorium, potassium and dose rates were presented in Fig. 4. These results can be compared with the world regulatory bodies on gamma radiation monitoring such as, the Nigerian Nuclear Regulatory Authority, Nuclear Safety and Radiation Protection, National Research Council, the United Nations Scientific Committee on the Effects of Atomic Radiation, International Atomic Energy Agency, International Commission on Radiological Protection, European Commission, World Health Organization and other bodies as list in [1]. The box-plot showing the comparison among the analyzed parameters are presented in Fig. 5. As revealed in this representation (Fig. 5), there is no outlier among the presented parameters. An outlier is an observation that is distant from other observations.

**Table 2a:** Descriptive statistics for the gamma radiation and dose rates (SET I).

Sample	∑N	Mean	SD	SEM	Variance	Sum	Skewness	Kurtosis	USS	CSS	CV	MAD
U-238	19	46.67	20.93	4.80	438.01	886.70	1.86	6.66	49267.82	7884.13	0.45	13.38
Th-232	19	71.76	10.31	2.37	106.298	1363.00	1.00	1.24	99740.62	1913.36	0.14	7.93
K-40	19	108.73	49.34	11.32	2434.90	2066.00	−0.14	−0.81	268435.06	43828.24	0.45	40.40
DR	19	67.86	12.21	2.80	148.98	1289.00	1.10	2.62	90184.92	2681.67	0.18	8.28

**Table 2b:** Descriptive statistics for the gamma radiation and dose rates (SET II).

Sample	∑N	GM	GSD	Mode	SW	Minimum	Imax.	Q1	Median	Q3	Maximum	Imax.	IR	Range
U-238	19	42.57	1.59	37.05	19	11.12	8	37.05	43.23	53.11	116.09	5	16.06	104.98
Th-232	19	71.10	1.14	73.49	19	58.06	8	62.93	73.08	76.73	98.25	13	13.80	40.19
K-40	19	95.15	1.79	125.20	19	31.30	14	62.60	125.20	156.50	187.80	2	93.90	156.50
DR	19	66.89	1.19	NA	19	46.15	8	61.48	67.67	72.53	101.22	5	11.05	55.08

**Fig. 6:**
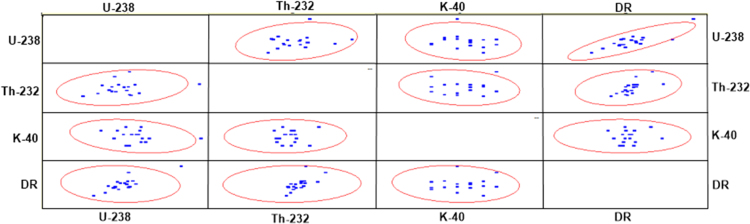
Scatter matrix or Scatter plot.

However, correlation analyses involving Pearson, Spearman, and Kendall correlations were employed in order to evaluate the degree of relationship that exist between or among the measured radiometric data. The correlations' results are shown in Table 3a, Table 3b, Table 3c. For the analyses, 2-tailed test of significance was used, and the results depict consistency among the three (3) analyses employed. In addition, the scatter matrix plot among the analyzed data was revealed in Fig. 6. Scatter matrix is a statistical technique that enables one to make estimates of the covariance matrix. The resulting Confidence Ellipse data sets produced from the radiometric data were attached as a supplementary file to this article. The confidence level for the Ellipse is 95%.

**Table 3a:** Correlation analyses of the radiometric data using Pearson correlations.

Parameters		U-238	Th-232	K-40	Dose rate
U-238	Corr.	1	0.23715	−0.15618	0.84124
	Sig.	–	0.32829	0.52315	6.39816E−6
Th-232	Corr.	0.23715	1	0.09765	0.69972
	Sig.	0.32829	–	0.69085	8.53511E−4
K-40	Corr.	−0.15618	0.09765	1	0.10123
	Sig.	0.52315	0.69085	–	0.68007
Dose rate	Corr.	0.84124	0.69972	0.10123	1
	Sig.	6.39816E−6	8.53511E−4	0.68007	–

2-tailed test of significance is used.

**Table 3b:** Correlation analyses of the radiometric data using Spearman correlations.

Parameters		U-238	Th-232	K-40	Dose rate
U-238	Corr.	1	0.20308	−0.19806	0.65525
	Sig.	–	0.40437	0.41633	0.00232
Th-232	Corr.	0.20308	1	0.06927	0.80509
	Sig.	0.40437	–	0.77812	3.21051E−5
K-40	Corr.	−0.19806	0.06927	1	0.13395
	Sig.	0.41633	0.77812	–	0.58458
Dose rate	Corr.	0.65525	0.80509	0.13395	1
	Sig.	0.00232	3.21051E−5	0.58458	–

2-tailed test of significance is used.

**Table 3c:** Correlation analyses of the radiometric data using Kendall correlations.

Parameters		U-238	Th-232	K-40	Dose Rate
U-238	Corr.	1	0.14243	−0.1209	0.49559
	Sig.	–	0.39967	0.49624	0.00324
Th-232	Corr.	0.14243	1	0.04441	0.64119
	Sig.	0.39967	–	0.80217	1.34482E−4
K-40	Corr.	−0.1209	0.04441	1	0.10722
	Sig.	0.49624	0.80217	–	0.54333
Dose rate	Corr.	0.49559	0.64119	0.10722	1
	Sig.	0.00324	1.34482E−4	0.54333	–

2-tailed test of significance is used.

Three (3) normality tests for statistical analysis involving Shapiro-Wilk, Lilliefors, and Kolmogorov-Smirnov were adopted in order to know whether the data sets are well-modeled by a normal distribution or not. The normality tests' results are presented in Table 4. The results showed that there is good fitting of a normal model to the radiometric data. For the three tests that were employed, the data was not significantly drawn from a normally distributed population at the 0.05 level.

**Table 4:** Normality tests using Shapiro-Wilk, Lilliefors, and Kolmogorov-Smirnov techniques.

Parameters	DF	Shapiro-Wilk		Lilliefors		Kolmogorov-Smirnov	
		Statistic	Prob<W	Statistic	Prob>D	Statistic	Prob>D
U-238	19	0.82565	0.00276	0.19911	0.04590	0.19911	0.38966
Th-232	19	0.91496	0.09135	0.16864	0.15299	0.16864	0.61001
K-40	19	0.93452	0.20968	0.15707	0.20000	0.15707	0.70943
Dose Rate	19	0.90163	0.05208	0.20572	0.03346	0.20572	0.35017

At the 0.05, the data was not significantly drawn from a normally distributed population.
